# Upper limb muscle overgrowth with hypoplasia of the index finger: a new over-growth syndrome caused by the somatic *PIK3CA* mutation c.3140A>G

**DOI:** 10.1186/s12881-018-0672-z

**Published:** 2018-09-04

**Authors:** Mohammad M. Al-Qattan, Ali Hadadi, Abdullah M. Al-Thunayan, Ahmed A. Eldali, Mohammed A. AlBalwi

**Affiliations:** 1 Division of Plastic Surgery, King Saud University, Riyadh, Saudi Arabia; 2 Division of Plastic Surgery, King Abdulaziz Medical City, Ministry of National Guard Health Affairs, Riyadh, Saudi Arabia; 3 King Saud bin Abdulaziz University for Health Sciences, College of Medicine, Riyadh, Saudi Arabia; 4 Department of Pathology & Laboratory Medicine, MC1122, King Abdulaziz Medical City, Ministry of National Guard Health Affairs, P.O Box 22490, Riyadh, 11426 Kingdom of Saudi Arabia; 5 Department of Medical Genomics Research, King Abdullah International Medical Research Center, Ministry of National Guard Health Affairs, Riyadh, Saudi Arabia

**Keywords:** Muscle, Overgrowth, PIK3CA, Somatic mutation, hypoplasia

## Abstract

**Background:**

Scientists have previously described an overgrowth syndrome in Saudi patients and named it ‘Upper limb muscle overgrowth with hypoplasia of the index finger’ syndrome.

**Case presentation:**

We describe a new case and document that the syndrome is caused by the somatic *PIK3CA* mutation c.3140A>G, p.His1047Arg. We also recruited one of the previously reported cases and found the same somatic mutation in the affected muscles. A wider classification of ‘PIK3CA-related pathology spectrum’ is presented which includes cancer, benign skin lesions/tumors, Cowden syndrome, isolated vascular malformations and various overgrowth syndromes. The latter entity is sub-divided into 3 sub-groups: overgrowth with brain involvement, overgrowth with multiple lipomatosis, and overgrowth without brain involvement/multiple lipomatosis.

**Conclusion:**

Our literature review indicated that “upper limb muscle overgrowth with hypoplasia of the index finger” is not as rare as previously thought to be. This overgrowth syndrome is unique and is caused by the somatic PIK3CA mutation c.3140A>G.

## Background

The term “*PIK3CA* – related overgrowth spectrum” (PROS) has gained popularity in recent literature to describe a group of overgrowth syndromes/disorders caused by somatic *PIK3CA* mutations [1]. In 2014, Al-Qattan described a new overgrowth syndrome in three unrelated Saudi patients and named it “upper limb muscle overgrowth in a proximo-distal gradient with hypoplasia of the index finger” (upper limb MO-hypoplasia IF). As the name implies, the two characteristic clinical features of the syndrome are:muscle overgrowth of one or both upper limbs which is manifested in a proximal-to-distal gradient, and congenital hypoplasia of the index finger. Other features of the syndrome are: flexion contractures of the fingers, hyper-extension deformity of the metacarpophalangeal joint of the thumb, ulnar deviation of the index finger, and a wide palm with abnormal palmar creases. In all previously reported cases, MRI confirmed that the limb overgrowth is due to enlarged/aberrant muscles with no fatty infiltration or vascular malformations. The most characteristic plain X-ray feature was widening of the space between the second and third metacarpals. No genetic analysis was done in Al-Qattan report [2].

In this paper, we describe a new case of upper limb MO-hypoplasia IF syndrome and document that it is caused by the somatic *PIK3CA* mutation c.3140A>G, p. His1047Arg. We also recruited one of the patients reported by Al-Qattan [2]; and confirmed the presence of the same mutation. A review of the literature was also done for similar cases. Finally, we offer a wider classification of “*PIK3CA* – related pathology spectrum”.

## Case presentation

### Clinical description - patient #1

**Fig. 1:**
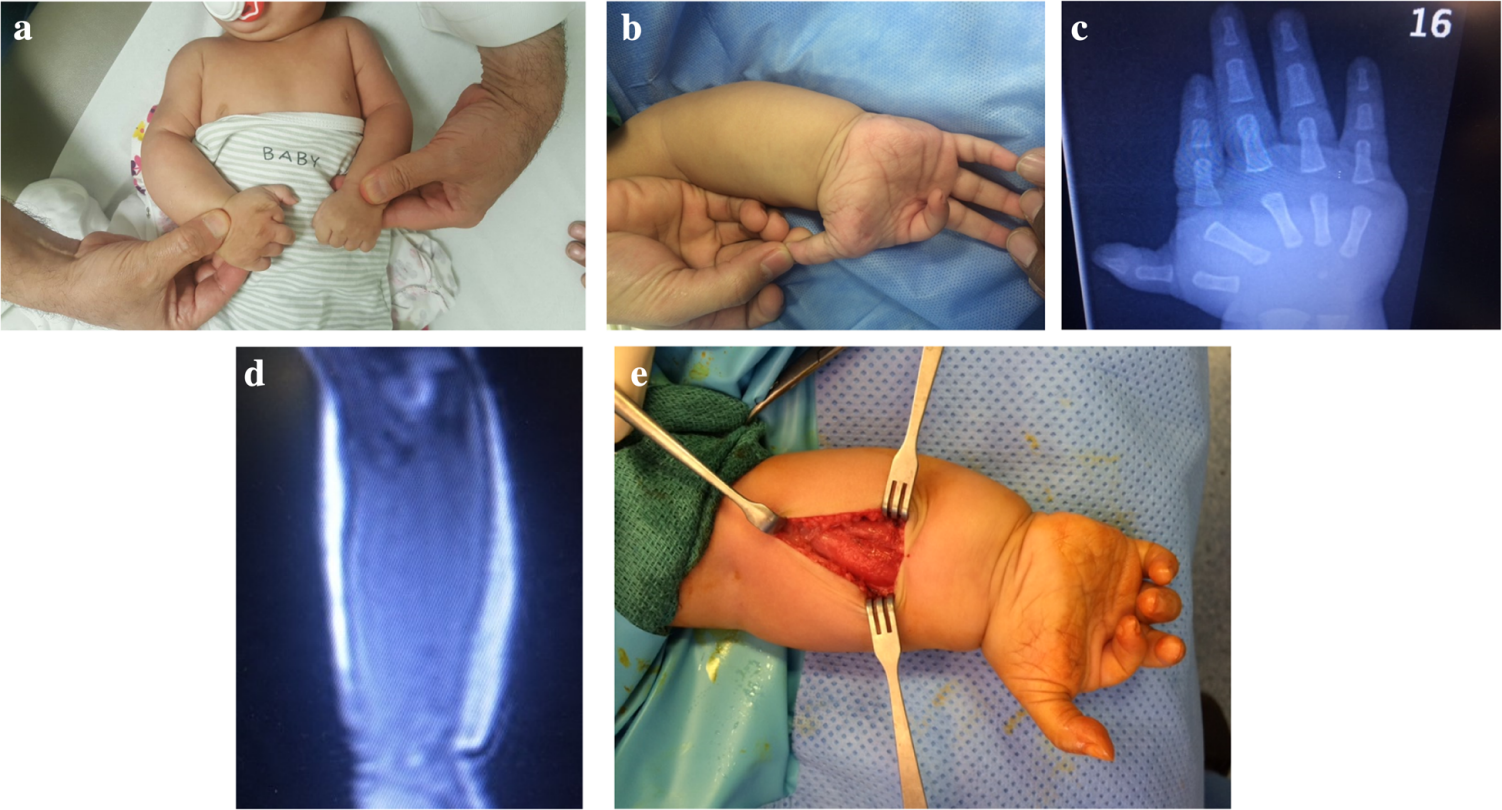
A 2-month-old boy with upper limb MO-hypoplasia IF syndrome. **a** A view to compare the affected right upper limb to the unaffected left upper limb. Note that the overgrowth is more prominent in the hand/forearm than the arm. **b** A view of the hand showing hypoplasia and ulnar-deviation of the index finger. Note the wide palm with abnormal palmar creases as well as the hyperextension of the thumb. **c** Plain X-ray of the hand showing a short hypoplastic index finger and widening of the space between the second and the third metacarpal. **d** MRI of the forearm showing the “pure” muscle overgrowth. **e** A view during the muscle biopsy procedure. Note the bulky muscles with a normal subcutaneous fat layer

A two –month old male infant presented to the surgical clinic with overgrowth of the right upper limb. He was born vaginally at term following a normal pregnancy. The birth weight and length were at the 50th centile. Family history was unremarkable. On physical examination there was overgrowth of the right upper limb which manifested in a proximo-distal gradient (Fig. 1a). The index finger was hypoplastic and ulnarly deviated (Fig. 1b). The palm was wide with abnormal palmar creases (Fig. 1b). There was also hyper-extension deformity of the metacarpophalangeal joint of the thumb (Fig. 1b). No other abnormalities were noted on clinical examination. Plain X-ray of the arm/ forearm showed no abnormalities. X-ray of the hand confirmed the hypoplasia of the index finger and widening of the space between the second and the third metacarpals (Fig. 1c). MRI confirmed that the overgrowth was due to enlarged muscles with no fatty infiltration or vascular malformations (Fig. 1d). A muscle biopsy was taken from the volar forearm muscles. During surgery, the subcutaneous fat was noted to be normal and the muscles appeared hypertrophied with no other abnormalities (Fig. 1e).

### Clinical description - patient #2

**Fig. 2:**
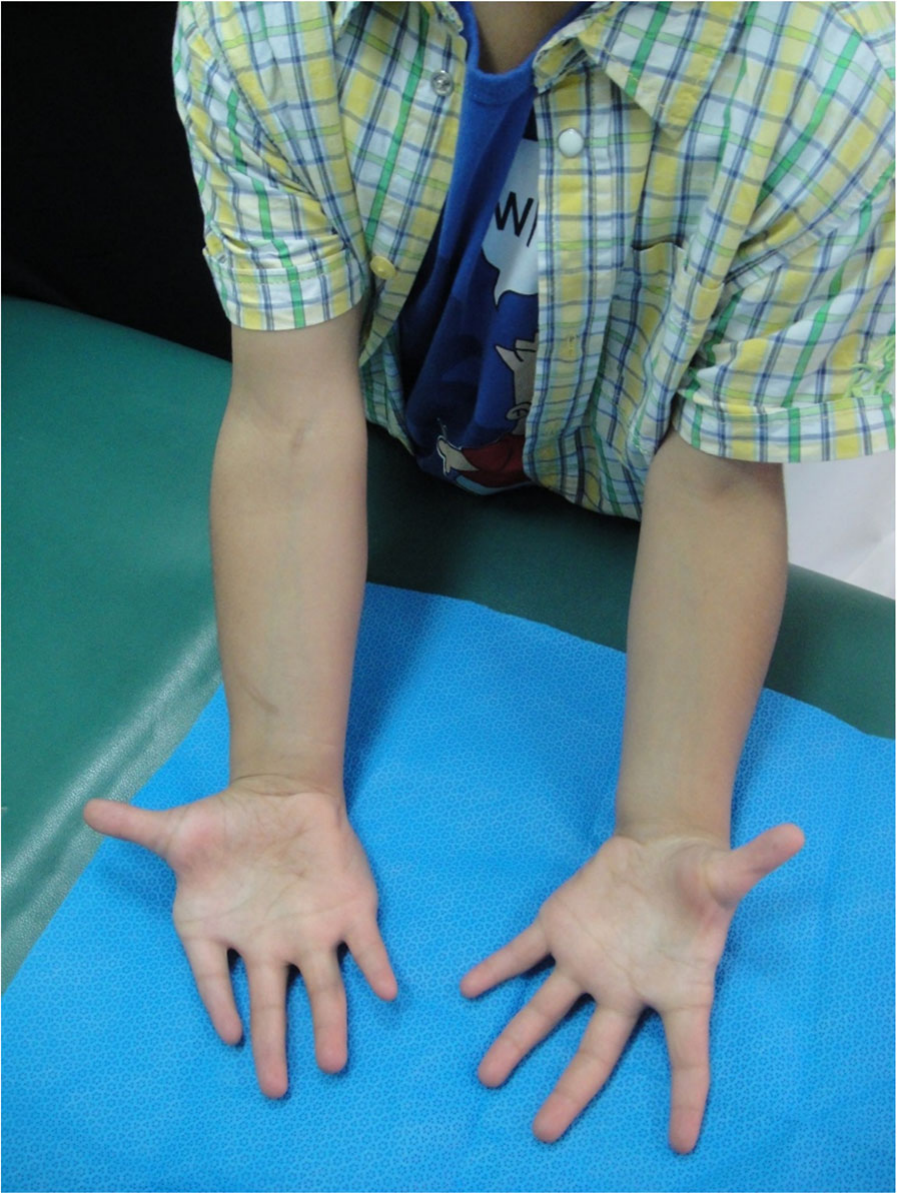
Patient #2 from Al-Qattan’s report [2] who underwent genetic analysis. The muscle overgrowth is bilateral but it is more pronounced on the right

We recruited case #2 reported by Al-Qattan in 2014 [2]. The 11-year old boy had muscle overgrowth of both upper limbs, but the involvement of the right upper limb was much more pronounced than the left (Fig. 2). The patient had all the classic clinical andradiological features of the upper limb MO – hypoplasia IF syndrome and showed no other abnormalities. A muscle biopsy was taken from the right forearm muscles and surgical findings were similar to our first case.

### Ethical approval and consents

Patients were assessed during their visit to the Plastic Surgery clinic. Detailed clinical data and radiological findings were obtained according to the Saudi Ministry of Health (MOH) guidelines. Protocols for peripheral blood sample collection, muscles biopsies, and genomic analysis were approved by the Research Committee at the National Hospital of Riyadh, Saudi Arabia in compliance withthe Declaration of HelsinkiPrinciples. Peripheral blood and muscle biopsy samples were obtained after signing a written informed consent form from all patients.

### PIK3CA mutational analysis

Genomic DNA was extracted from the collected peripheral blood samples and muscle biopsies using Qiagen DNeasykit (Qiagen, Germany) according to the manufacturer’s instructions. The region, exons and the intron/exons junctions of the *PIK3CA* gene were amplified by PCR and bidirectional sequences were performed using previously described primers byBachman et al. [3] using an ABI prism Big Dye Terminator v. 3.1 cycle sequencing kit (Applied Biosystems, Foster City, USA) and an ABI 3130 Genetic Analyzer sequencer (Applied Biosystems, Foster City, USA). Mutationswere identified by comparison to reference sequences (GenBank Accession No NM_006218.3; http://www.ncbi.nlm.nih.gov) using CLC Genomics Workbench v 8.0 (CLC bio, Aarhus, Denmark) and these were checked against theupdatedSingle Nucleotide PolymorphismsDatabase (dSNPs; http://www.ncbi.nih.gov) and the Catalogue Of Somatic Mutation In Cancer (COSMIC, https://cancer.sanger.ac.uk). Furthermore, analysis of the mutation was evaluated using different bioinformatics tools including ‘Mutation Taster’ (http://www.mutationtaster.org) to predict confirmation of “disease causing” mutation.

## Discussion and conclusions

**Fig. 3:**
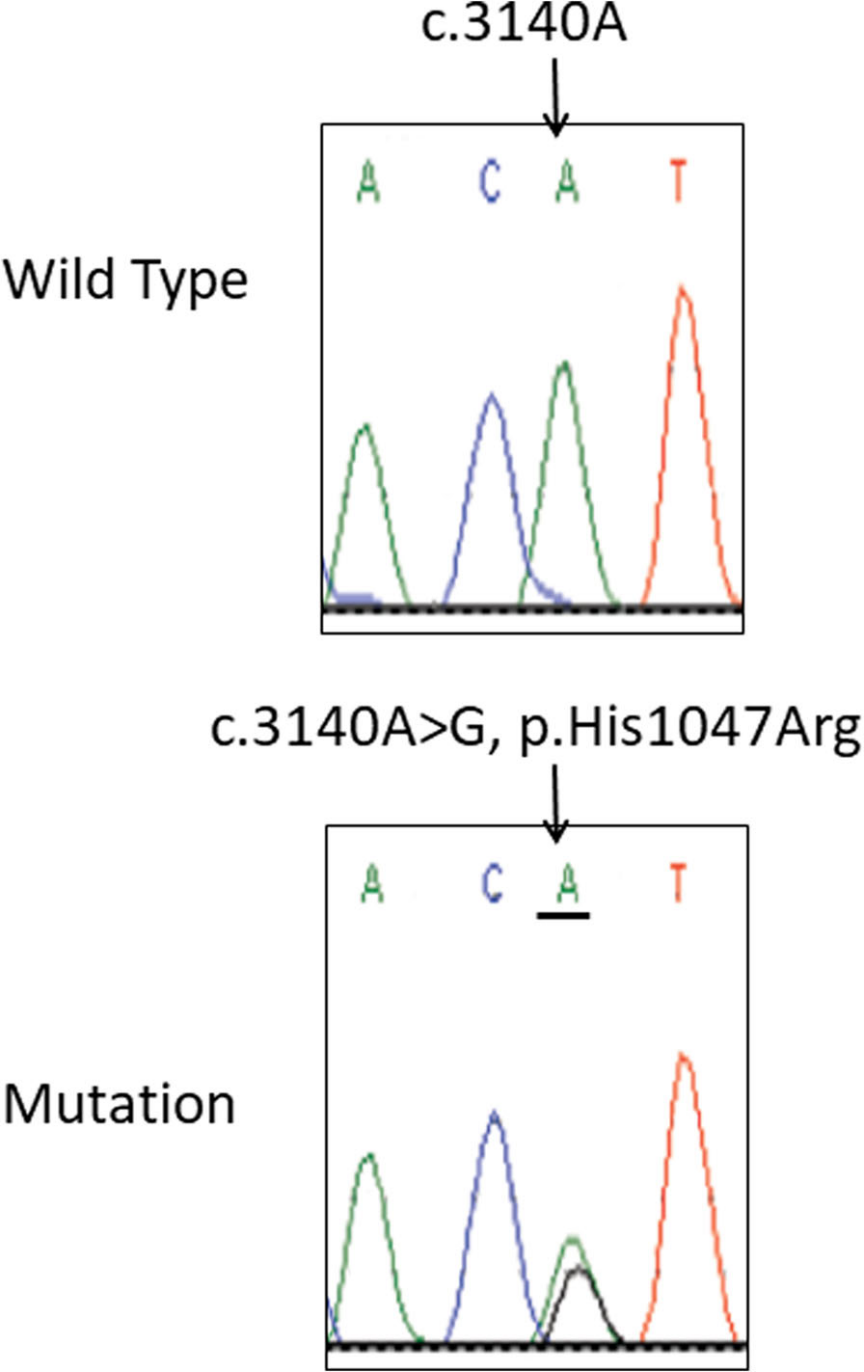
Examples of chromatograms obtained from patients’ blood (top) and muscle biopsies (bottom). The arrow indicates the somatic mutation (bottom) nucleotide and amino acid change within the *PIK3CA* gene at exon20 (c.3140A>G, p.His1047Arg)

Clinically, both of our patients had all the characteristic features of theupper limb MO-hypoplasia IF syndrome.Histologically, muscle biopsies from both patientsshowed hypertrophic muscle fibers with no fatty infiltration or vascular malformations.Molecular genetic analysis of both patients showed a similar somatic mutation within *PIK3CA* gene at exon20 (c.3140A>G, p. His1047Arg) in the genomic DNA obtained from the muscle biopsies (Fig. 3), but not from blood leucocytes.

The upper limb MO-hypoplasia IF syndrome is not as rare as previously thought. Upon reviewing the English literature prior to Al-Qattan [2] report, several similar cases were found in the Hand Surgery literature [4–9]. These hand surgeons commented on the muscle hypertrophy of the upper limbs as well as the aberrant muscles and the deviation of the fingers. However, when we examined the illustrations of these reports, we also found that hypoplasia of the index finger was a constant feature. We also found two similar cases reported in the Genetics literature. Castiglioni et al. [10] reported on a 6-year old girl with unilateral isolated upper limb muscle overgrowth. The authors only commented on the ulnar drift of the index finger; although the illustrations also showed hypoplasia of the index finger. An open biopsy of the interosseous muscle was done and revealed the same somatic mutation in *PIK3CA* (p.3140A>G, p. His1047Arg). Rasmussen et al. [11] reported on a 20-year old female with bilateral upper limb muscle hypertrophy and concurrent upper back lipoma. The authors only commented on the overlapping of the index fingers over the middle fingers; although the illustrations also showed hypoplasia of both index fingers. A muscle biopsy also revealed the same somatic mutation in *PIK3CA*. Our review clearly confirms that the “upper limb MO-hypoplasia IF” syndrome is a unique entity that should be added to the PROS. The fact that all cases that underwent genetic analysis showed the exact same somatic mutation in *PIK3CA* is interesting. However, this does not mean that a genotype-phenotype correlation exists; since 70% of patients with PROS have one of three somatic mutations in *PIK3CA*: p. Glu542Lys, p. Glu545Lys, and p. His1047Arg [12]. These three hotspot mutations also make about 70% of *PIK3CA* somatic mutations associated with cancer [13].

We believe that a wider classification of “*PIK3CA*-related pathology spectrum” (PRPS) should be offered (Table 1) and should include somatic and germline mutations. This pathology spectrum should include cancer, benign skin lesions/ tumors, Cowden syndrome, isolated vascular malformations as well as the PROS [1, 12–25].The PROS should also be extended to include the “upper limb MO-hypoplasia IF” as well as the Klipple-Trenaunay syndrome as described by Yeung et al. [12]. We also recommend dividing the PROS into three sub-groups: PROS with brain involvement, PROS with multiple lipomatosis, and PROS without brain involvement / multiple lipomatosis. Our PRPS is summarized (Table 1). Note that our classification is based on pathological entities (which is more clinically oriented) and not based on genotype-phenotype correlations proposed in Mirzaaet al. [26].

**Table 1:** *PIK3CA*-Related Pathology Spectrum (PRPS)

Pathology	Comments	Reference
Cancers	Several cancers are known to be associated with *PIK3CA* somatic mutations such as cancer of the breast, colon, stomach, liver, lung and ovaries	[13]
Benign skin lesions/ Tumors	Seborrheic keratosis, epidermal nevi, Lichenoid keratosis, hydradenomapapilliferum are associated with *PIK3CA*somaticmutations	[1, 12, 14, 15]
Cowden syndrome type5	Cowden syndrome (multiple hamartomas of the mucous membrane and skin lesions) is put in a separate group because it is associated with *PIK3CA* germline and not somatic mutations	[16]
Isolated vascular malformations	Isolated lymphatic, venous and lymphatico-venous malformation are associated with *PIK3CA* somatic mutations.	[12, 17]
PROS (*PIK3CA* – Related overgrowth spectrum) with brain involvement	MCAP (Megalencephaly, capillarymalformation*,* polymicrogyria*,* syndactyly*,* polydactyly)*,* MPPH (Megalencephaly polymicrogyria, polydactyly, hydrocephalus)*,* DMEG (Dysplastic megalencephaly), Hemi-megalencephaly, and Focal cortical dysplasiaare associated with *PIK3CA* somatic mutations	[1, 18, 19]
PROS with multiple lipomatosis as the main presenting feature	HHML (Hemihyperplasia-multiple lipomatosis), facial infiltrating lipomatosis (facial lipomatosis, skeletal overgrowth, macrodontia, hemi-macroglossia, oral mucosal neuromas), mesenteric lipomatosis (Mesenteric lipomatosis, and insulin hypersensitivity are the main presenting features. Although the skin may have subcutaneous lipomas, there is relative lack of subcutaneous adipose tissue), multiple subcutaneous lipomatosis-scoliosis-multiple internal organ lymphatic malformations described by Yeung et al. [12]. All of these disorders are associated with *PIK3CA* somatic mutations	[1, 12, 18, 20, 21]
Other PROS disorders	CLOVES syndrome (Congenital, lipomatous overgrowth, vascular malformations, epidermal nevi and skeletal/spinal anomalies.Splayed feet with macrodactyly and wide sandal gap are characteristic), fibro-adipose overgrowth (FAO, progressive segmental overgrowth of subcutaneous, muscular and visceral fibro-adipose tissue. Skeletal overgrowth of the lower limb with macrodactyly of the feet are characteristic), isolated macrodactyly of the hands/feet, nerve-oriented macrodactyly (known in the hand surgery literature as lipo-fibromatous hamartoma of nerve with macrodactyly because the macrodactyly is seen in the digital rays supplied by the affected nerve). Rios et al. [24] reported on a case with nerve -oriented-macrodactyly and concurrent muscle hypertrophy. Klippel-Trenaunay syndrome (progressive growth of the lower limb with cutaneous capillary malformations and deep vascular malformations), and upper limb MO - hypoplasia IF are also included in this group. All of these disorders are associated with *PIK3CA* somatic mutations	[1, 12, 18, 22–25] and the current report

**Fig. 4:**
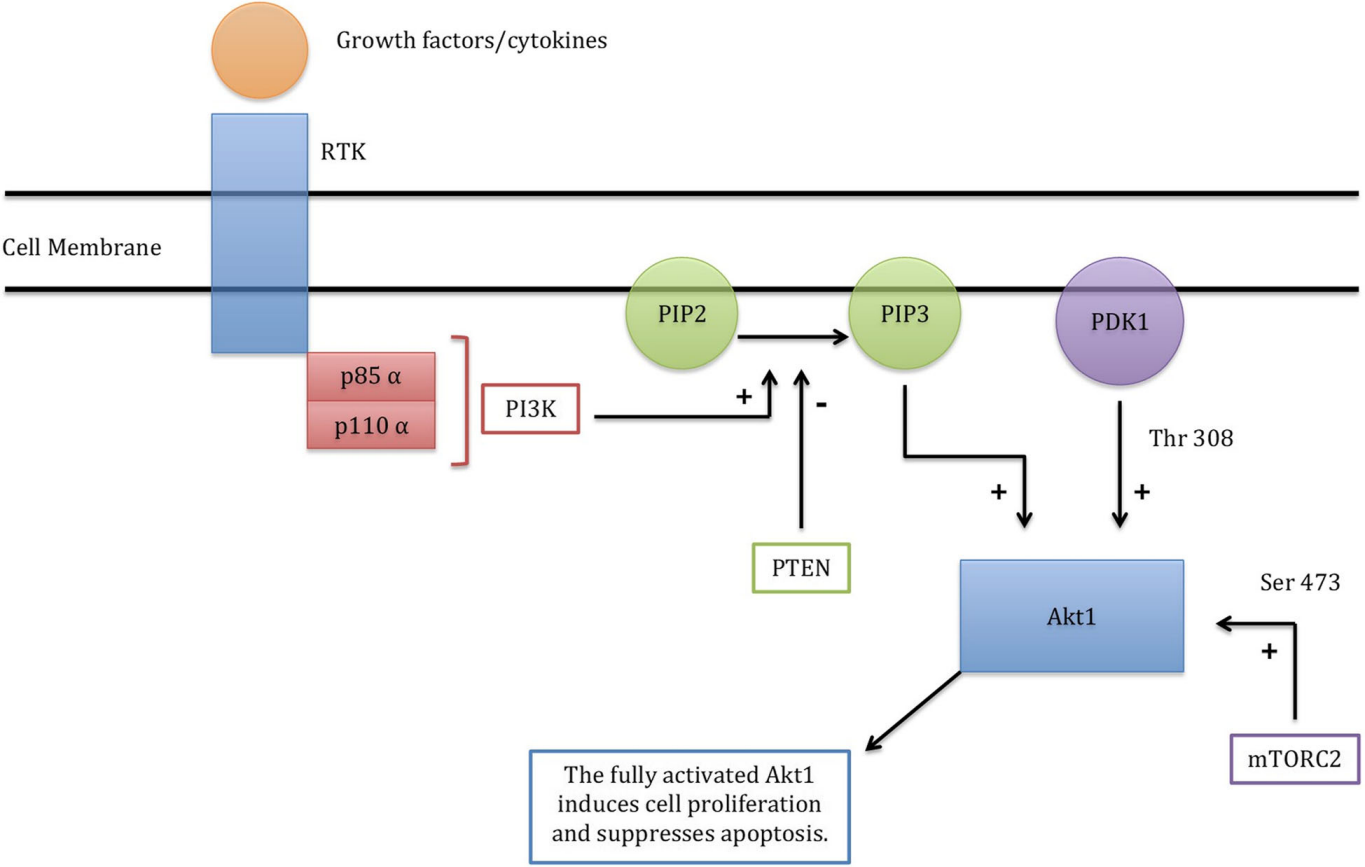
The PI3K pathway. The RTK (receptor tyrosine kinase) is stimulated by growth factors and cytokines. This will activate the PI3K. The activated PI3K will convert PIP2 to PIP3 at the cell membrane. The PIP3 will recruit Akt1 (protein kinase B) to the plasma membrane; allowing PDK1 to activate Akt1 at Thr 308. Further activation of Akt1 at Ser 473 is done by m-TCRC2 (mechanistic Target of Rapamycin Complex – 2). The fully activated Akt1 will then induce cell proliferation and will suppress apoptosis. The pathway is negatively regulated by PTEN which converts PIP3 into PIP2.

The *PIK3CA* gene encodes the p110 α catalytic sub-unit of PI3K (phosphatidylinositol 3-kinase). All somatic and germline mutations of the PRPS are activating (gain-of-function) mutations of the PI3K pathway leading to enhanced cellular proliferation. The PI3K pathway (Fig. 4) is initiated by growth factor/cytokine stimulation of a receptor tyrosine kinase in the plasma membrane [27]. This activates PI3K (which is heterodimer composed of a regulatory sub- unit known as p85α and a catalytic sub-unit known as p110α). The activated PI3K will then add a phosphate group to PIP2 to become PIP3 (phosphatidyl inositol bis-phosphate to phosphatidyl inositol tris-phosphate) at the plasma membrane lipids. The PIP3 will then recruit Akt1 (protein kinase B) to the plasma membrane, allowing PDK1 (phosphoinositide- dependent kinase 1) to phosphorylate and activate Akt1 at its activation loop site (Thr 308). Further activation of Akt1 requires its phosphorylation at another site (Ser 473) by mTORC2 (mechanistic Target of Rapamycin Complex-2). The fully activated Akt1 will then induce several cellular processes including stimulation of cell proliferation and suppression of apoptosis. The PI3K pathway is negatively regulated by PTEN (Phosphate and tensin homolog) and this is done by converting PIP3 into PIP2. Hence gain-of-function of *PIK3CA*, *AKT1*, and MTOR; or loss-of-function of *PTEN* will result in cancer, benign tumors, and overgrowth syndromes. For example, proteus syndrome (sporadic, mosaic, asymmetric and progressive overgrowth of body parts with cerebriform connective tissue nevi being characteristic of the syndrome) may be caused by gain-of-function somatic mutations of *AKT-1* [28], or loss-of-function germline mutations of *PTEN* [29].Similarly, Cowden and Cowden-like syndromes are caused by loss-of-function germline *PTEN* mutations as well as gain-of-function germline mutations of *PIK3CA* and *AKT1* [16]. Finally, megalencephalysyndromes maybe caused by gain-of-function somatic mutations of *PIK3CA* (Table 1) or gain-of-function germline mutations of *MTOR* [30].

In conclusion, we describe a unique form of overgrowth syndrome caused by the *PIK3CA* somatic mutation: p. His1047Arg. We also review similar cases in the literature and offer a wider classification of *PIK3CA* – related pathology spectrum.

## Abbreviations

MOH: Saudi Ministry of Health
PROS: *PIK3CA* related overgrowth spectrum
PRPS: *PIK3CA*-Related Pathology Spectrum
